# Dosage Related Efficacy and Tolerability of Cannabidiol in Children With Treatment-Resistant Epileptic Encephalopathy: Preliminary Results of the CARE-E Study

**DOI:** 10.3389/fneur.2019.00716

**Published:** 2019-07-03

**Authors:** Richard J. Huntsman, Richard Tang-Wai, Jane Alcorn, Stephanie Vuong, Bryan Acton, Scott Corley, Robert Laprairie, Andrew W. Lyon, Simona Meier, Darrell D. Mousseau, Doris Newmeyer, Erin Prosser-Loose, Blair Seifert, Jose Tellez-Zenteno, Linda Huh, Edward Leung, Philippe Major

**Affiliations:** ^1^Cannabinoid Research Initiative of Saskatchewan, University of Saskatchewan, Saskatoon, SK, Canada; ^2^Department of Pediatrics, Royal University Hospital, University of Saskatchewan, Saskatoon, SK, Canada; ^3^Division of Child Neurology, Department of Pediatrics, Loma Linda University, San Bernardino, CA, United States; ^4^College of Pharmacy and Nutrition, University of Saskatchewan, Saskatoon, SK, Canada; ^5^Saskatchewan Health Authority and Department of Psychology, University of Saskatchewan, Royal University Hospital, Saskatoon, SK, Canada; ^6^Clinical Trial Support Unit, Royal University Hospital, University of Saskatchewan, Saskatoon, SK, Canada; ^7^Department of Pathology and Laboratory Medicine, Royal University Hospital, Saskatchewan Health Authority, Saskatoon, SK, Canada; ^8^Cell Signalling Laboratory, Departments of Psychiatry and Physiology, University of Saskatchewan, Saskatoon, SK, Canada; ^9^Department of Pharmaceutical Services, Royal University Hospital, Saskatchewan Health Authority, Saskatoon, SK, Canada; ^10^Division of Neurology, Department of Medicine, Royal University Hospital, University of Saskatchewan, Saskatoon, SK, Canada; ^11^Division of Pediatric Neurology, Department of Pediatrics, BC Children's Hospital, University of British Columbia, Vancouver, BC, Canada; ^12^Division of Pediatric Neurology, Department of Pediatrics, Children's Hospital, University of Manitoba, Winnipeg, MB, Canada; ^13^Service de Neurologie Pédiatrique, Département de Neurosciences, Centre Hospitalier Universitaire Sainte-Justine, Université de Montréal, Montreal, QC, Canada

**Keywords:** cannabidiol, Δ^9^-tetrahydrocannabinol, cannabis, epileptic encephalopathy, cannabinoid plasma levels

## Abstract

**Purpose:** There is uncertainty regarding the appropriate dose of Cannabidiol (CBD) for childhood epilepsy. We present the preliminary data of seven participants from the Cannabidiol in Children with Refractory Epileptic Encephalopathy (CARE-E) study.

**Methods:** The study is an open-label, prospective, dose-escalation trial. Participants received escalating doses of a *Cannabis* Herbal Extract (CHE) preparation of 1:20 Δ^9^-tetrahydrocannabinol (THC): CBD up to 10–12 mg CBD/kg/day. Seizure frequency was monitored in daily logs, participants underwent regular electroencephalograms, and parents filled out modified Quality of Life in Childhood Epilepsy (QOLCE) and Side Effect rating scale questionnaires. Steady-state trough levels (C_ss, Min_) of selected cannabinoids were quantified.

**Results:** All seven participants tolerated the CHE up to 10–12 mg CBD/kg/day and had improvements in seizure frequency and QOLCE scores. C_SS, Min_ plasma levels for CBD, THC, and cannabichromene (CBC) showed dose-independent pharmacokinetics in all but one participant. C_SS, Min_ CBD levels associated with a >50% reduction in seizures and seizure freedom were lower than those reported previously with purified CBD. In most patients, C_SS, Min_ levels of THC remained lower than what would be expected to cause intoxication.

**Conclusion:** The preliminary data suggest an initial CBD target dose of 5–6 mg/kg/day when a 1:20 THC:CBD CHE is used. Possible non-linear pharmacokinetics of CBD and CBC needs investigation. The reduction in seizure frequency seen suggests improved seizure control when a whole plant CHE is used. Plasma THC levels suggest a low risk of THC intoxication when a 1:20 THC:CBD CHE is used in doses up to 12 mg/kg CBD/kg/day.

## Introduction

Recent trials with pharmaceutical grade cannabidiol (CBD) or CBD-enriched *Cannabis* Herbal Extract (CHE) support CBD's ability to reduce seizure frequency in children with intractable epilepsy, including those with epileptic encephalopathy (1–5). Yet, there are significant knowledge gaps regarding the use of CBD and other cannabinoids in children, including the pharmacokinetics (PK), pharmacogenetics, and dose-concentration-effect relationships for these compounds (6). The resultant inability to provide evidence-based dosing and therapeutic monitoring of *Cannabis*-based products in children, combined with concerns regarding potential intoxicant effects of Δ^9^-tetrahydrocannabinol (THC), leads to a reluctance by many physicians to authorize CHE to these patients.

The age-related developmental changes that influence drug PK and pharmacodynamics (PD) complicate the development of appropriate dosing regimens for pediatric age groups (6). Without an understanding of dose concentration-effect relationship, a dosing regimen is largely empirical and/or anecdotal, and fraught with potential safety concerns.

CARE-E is a multi-center, phase 1, open-label, dosage escalation study using a Health Canada approved and Good Manufacturing Practices certified 1:20 THC:CBD CHE as adjunct therapy to treat children with epileptic encephalopathy. The primary objectives were to assess the safety and efficacy of CBD-enriched CHE, whereas secondary objectives included an analysis of trough steady state (C_SS, Min_) levels of CBD, THC, and cannabichromene (CBC); as well as an assessment of the correlation between cannabinoid levels and therapeutic effect. CBC levels were measured as the CHE used in this study contained 4% CBC by volume. We present results for seven CARE-E participants recruited at the University of Saskatchewan site.

## Methods

### Trial Design

The study is a phase 1, open-label, dosage-escalation clinical trial in which participants receive a 1:20 THC:CBD CHE in twice daily dosing. Upon enrollment (Visit 1) participants continue their current anticonvulsant regimen and baseline seizure frequency is determined for 1 month. At Visit 2 CHE dosing is initiated with a CBD dose of 2–3 mg/kg/day. At Visits 3–5 the CHE is increased at 1-month intervals with CBD doses of 5–6 mg/kg/day at Visit 3, 8–9 mg/kg/day at Visit 4, and 10–12 mg/kg/day at Visit 5. At Visit 6 the CHE is weaned over a 1-month period after which the participants have their end of study visit (Visit 7). Care-givers monitor and record seizure frequencies in daily seizure logs. The complete study design and methodology have been described previously (7).

### Ethics

Prior to enrollment, written and informed consent was obtained from the child's parents or legal guardian. This study received a No Objection Letter (NOL) from Health Canada, was approved by the University of Saskatchewan Biomedical Research Ethics Board and registered with ClinicalTrials.gov (NCT03024827).

### Participants

Inclusion criteria included pediatric patients between the ages of 1 to 10 years with epileptic encephalopathy resistant to standard medical treatment (as per International League Against Epilepsy definition of drug resistant epilepsy) and at minimum one major seizure per week or four major seizures per month (8). Seven participants from Saskatoon who met the inclusion criteria completed the study. All study data were collected and managed using the REDCap electronic data capture tool hosted at the University of Saskatchewan (9).

### Efficacy Outcome Measures

Seizures occurring in a cluster were counted as a single seizure due to challenges arising from caregivers individually recording each seizure within a cluster. The data from the seizure logs was entered into REDCap (9) at each visit and underwent an independent audit performed by the University of Saskatchewan Clinical Trial Support Unit. To allow for variations in the length between study visits, the average daily number of seizures between visits was calculated by dividing the number of seizures recorded between each visit by the number of days between visits.

At Visits 2–6, participants underwent a 2-h EEG for assessment of degree of background slowing and spike index. The first EEG was performed prior to starting the CHE and each subsequent EEG was performed prior to a scheduled CHE dosage increase. To ensure consistency in EEG interpretation, an EEG rating scale for background slowing (encephalopathy) proposed by Lüders was used (10). A spike index ranked on a five-point scale ranging from 0 (= No Spikes) to 4 (= Continuous Spiking, defined as spikes occupying more than 70% of the EEG) was also calculated for each EEG.

At Visits 2–7, parents completed a modified Quality of Life in Childhood Epilepsy (QOLCE-55) survey which, in addition to questions assessing the domains including cognition, physical independence, social engagement, well-being, behavior (11), contained 13 additional items about sleep, verbal and non-verbal communication, interpersonal interactions, and irritability. Each item was rated from 1 (= Very Often) to 5 (= Never) or marked “Not Applicable.” The scores for reverse items were inverted and then all scores were transformed using (Score-1) × 25. The mean score for each subscale was calculated ignoring those marked “Not Applicable.”

### Safety Outcome Measures

During the study caregivers recorded a description all adverse events associated with CHE in a participant diary. From Visit 3 to Visit 7, caregivers rated adverse effects previously described with CBD. Sleepiness/Lethargy and Irritability were rated from 0 (= Not Present) to 4 (= Present All The Time). Nausea/Vomiting and Diarrhea were rated from 0 (= Not Present) to 5 (= More Than Once Per Day). At each visit, the information for the preceding month was self-reported and provided to the study nurse.

At Visits 2–6, blood samples were collected for complete cell count and differential cell count, sodium, potassium, chloride, calcium, magnesium, phosphate, creatinine, urea, aspartate transaminase, alanine transaminase, alkaline phosphatase, and gamma glutamyltransferase, total and direct bilirubin, lipase, albumin, cholesterol, and triglycerides. Elevations in liver enzymes or lipase were considered significant if they were more than three times the upper limit of the normal reference range.

### Quantification of Cannabinoids in Plasma and Steady State Trough Levels (C_SS, Min_)

To measure plasma trough steady-state (C_SS, Min_) cannabinoid levels, blood was collected on Visits 2–5 into lithium heparin Barricor vacutainers and centrifuged for 10 min in a clinical centrifuge (1,500 rpm) (12). These plasma samples (200 μL) were prepared and analyzed for CBD, CBC, and THC levels according to a validated liquid chromatography-mass spectrometry (LC-MS/MS) method that while developed independently in our lab is similar to a previously reported validated plasma cannabinoid assay (7, 13). All samples were stored at −70°C prior to analysis. Analytical method validation indicated the assay was specific and linear from 0.49 to 125 ng ml^−1^, for THC and CBD, and 0.98–125 ng ml^−1^ CBC with *r*^2^ > 0.998. Matrix effects ranged from 40 to 50% depending upon analyte resulting in extraction efficiencies in a similar range but recoveries were >88%. Intra- and inter-day precision and accuracy of the method was within ± 15%. The samples were analyzed in three batches (October 2017, May 2018, and March 2019). While most samples were analyzed within 3 months of collection, some were analyzed up to 8 months after collection. Stability analysis indicates stability of cannabinoids stored frozen for 3 months but stability beyond this is unknown. Full details of the quantification method of cannabinoids in plasma samples are available as Supplementary Protocol provided with this manuscript.

### Steady-State Trough Anticonvulsant Levels

During the study, the participants' anticonvulsant medications were not adjusted. The exception was clobazam, which was decreased if it was felt that clobazam side effects were being exacerbated by the known interaction between CBD and clobazam (14). Prior to decreasing the dose of clobazam, trough clobazam, and norclobazam levels were measured.

Trough anticonvulsant levels were measured at Visits 2–6 to identify a possible drug interaction with CBD, a known competitive inhibitor of CYP2C and CYP3A isozymes (15). C_SS, Min_ levels were obtained for valproic acid, lamotrigine, levetiracetam, topiramate, and clonazepam. C_SS, Min_ levels for stiripentol were not obtained as this assay was not available to us through the Saskatchewan Provincial Health Laboratory or its partnering laboratories.

### Statistical Analysis

**Figure 1:**
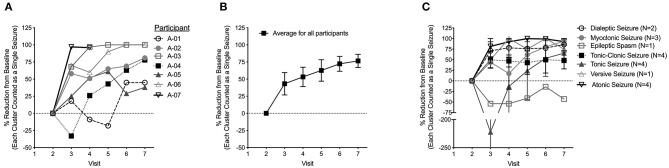
**(A)** Percentage reduction in daily average seizure frequency as compared to baseline. The value shown at each visit represents the decrease in seizure frequency from baseline during the preceding month. **(B)** Average percentage reduction in daily average seizure frequency from baseline for all seven participants at each study visit. **(C)** Average percentage reduction in daily seizure frequency from baseline for all seven participants broken down into seizure type. Data are shown as mean ± s.e.m.

**Figure 2:**
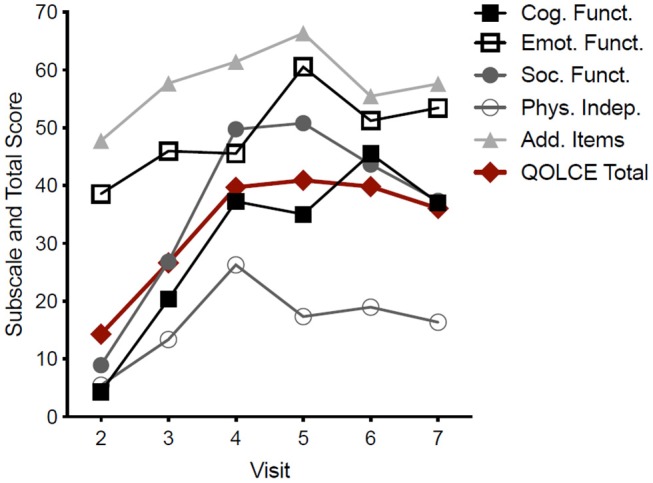
Pooled QOLCE-55 scores and subscores for all seven participants. The values shown at each visit represent the QOLCE-55 total and subscores for the preceding month. Data are mean from seven participants.

**Figure 3:**
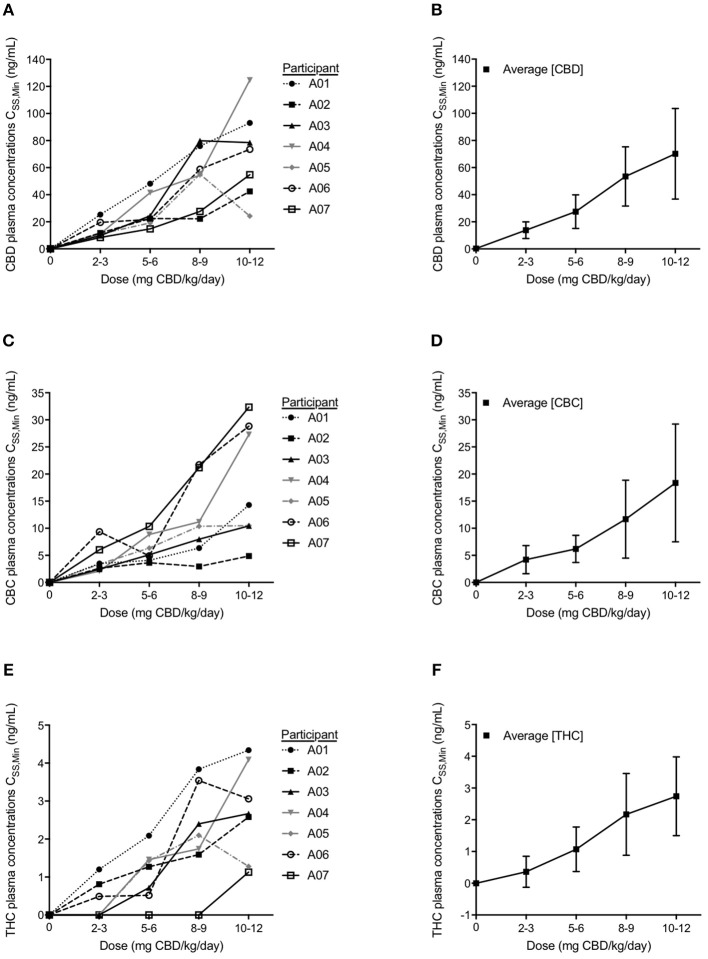
Participant minimum steady state (C_SS,Min_) plasma concentrations and average plasma C_SS,Min_ levels for each cannabinoid of cannabidiol (CBD) **(A,B)**, cannabichromene (CBC) **(C,D)**, and Δ^9^-tetrahydrocannabinol (THC) **(E,F)** analyzed with LC-MS/MS. Values shown represent steady state levels after 1 month on the corresponding dosage of CBD measured just prior to a dose administration. Data are mean ± s.e.m.

Due to the small sample size reported a formal statistical analysis was not performed. Data are presented from individual participants and as the mean ± standard error of the mean (s.e.m.) (Figures 1, 3) and in a descriptive manner to illustrate trends emerging from the data (Figure 2). A formal analysis will be done when all patients are included in the trial.

## Results

### Demographic Characteristics and Compliance

At time of enrolment, all participants failed at least 2 appropriate anticonvulsants, and none were using the ketogenic diet or had a vagal nerve stimulator. All participants were fully compliant with all study protocols. Table 1 summarizes study participant characteristics. As per the publishing guidelines of this journal the participants' gender is not included and age at recruitment is provided in ranges (1–3, 4–6, 7–10 years).

**Table 1:** Participant characteristics at time of recruitment into CARE-E including age, epilepsy diagnosis, and concomitant anticonvulsant medications.

**Participant ID**	**Age (years)**	**Weight (kg)**	**Epilepsy diagnosis**	**Predominant seizure types**	**Number of seizures in baseline month**	**Concomitant anticonvulsant therapies and daily dosage (mg/kg/day)**
A-01	4–6	17.2	Dravet syndrome (SCN1A mutation)	Tonic-clonic, tonic, myoclonic	11	Stiripentol (50 mg/kg/day) Clobazam (1.3 mg/kg/day)
A-02	4–6	14.6	Dravet syndrome (SCN1A mutation)	Dialeptic, myoclonic (in clusters), Tonic-Clonic	343	Clobazam (0.3 mg/kg/day) Stiripentol (63 mg/kg/day) Topiramate (20 mg/kg/day)
A-03	4–6	23.7	Lennox Gastaut syndrome, continuous spike wave in sleep (evolved from cryptogenic infantile spasms)	Dialeptic, atonic, tonic	195	Valproic Acid (29 mg/kg/day) Clobazam (1.2 mg/kg/day) Lamotrigine (5.3 mg/kg/day) Levetiracetam (59 mg/kg/day)
A-04	1–3	11.4	Lennox Gastaut syndrome (Cerebral palsy-perinatal asphyxia)	Epileptic spasms, tonic, myoclonic	1,223	Lamotrigine (4 mg/kg/day) Valproic Acid (53 mg/kg/day)
A-05	1–3	14.3	Lennox Gastaut syndrome (cerebral palsy-perinatal asphyxia)	Atonic (in clusters), tonic, tonic-clonic	56	Lamotrigine (10.2 mg/kg/day) Clonazepam (0.08 mg/kg/day)
A-06	7–10	20.9	Dravet syndrome (SCN9A mutation)	Tonic clonic	10	Topiramate (9.6 mg/kg/day), Clonazepam (0.17 mg/kg/day) Valproic Acid (36 mg/kg/day)
A-07	4–6	18.6	Dravet syndrome (SCN1A mutation)	Atonic, tonic clonic, versive partial	165	Valproic Acid (19 mg/kg/day) Clobazam (1.1 mg/kg/day) Stiripentol (29 mg/kg/day)

*Underlying aetiology of epileptic encephalopathy listed in parentheses*.

### Safety and Tolerability Outcome Measures

While all participants reported Sleepiness/Lethargy and Irritability during the study, no scores increased by more than two points. Irritability improved in two participants following a decrease in clobazam dosage. Occasional incidences of nausea and vomiting, diarrhea, increased appetite, difficulty sleeping and spasticity were reported. Changes in the side-effect rating scales were not consistent and, apart from nausea and vomiting, did not correlate with increased doses of the CHE. None of the side effects were severe enough to prompt withdrawal from the study. The side effects rating scale scores are provided in Supplementary Tables 1A–D.

No significant changes in complete blood count and differential, electrolytes, renal panels, triglyceride, cholesterol, albumin, or bilirubin levels were observed. All participants had elevated ALP at Visit 1; however, these levels did not increase with the introduction and titration of CHE, with the exception of participant A-07, whose ALP increased to 300 U/L (reference: 30–110 U/L) at Visit 4, but decreased back to 144 U/L at Visit 5.

Participant A-01 had a slight elevation of GGT at 44 U/L (reference 10–35 U/L) seen at Visit 3 only. Participant A-03 had a marked elevation of GGT to 738 U/L (10–50 U/L) during an admission to Pediatric Intensive Care for sepsis. GGT decreased to 73 U/L the following month and returned to normal on post-study follow up despite continuing CHE.

Participant A-04 had slight elevations of AST at Visits 3 and 6 (48 U/L and 44 U/L, respectively -reference: 10–40 U/L). GGT was elevated prior to, and remained elevated throughout, the study, reaching a peak of 88 U/L at Visit 4. Participant A-04's serum lipase at 173 U/L (normal: 22–51 U/L) was significantly elevated at Visit 5. As he was asymptomatic and an abdominal ultrasound was normal, he continued to receive CHE. By Visit 6, lipase levels decreased to 83 U/L and returned to normal following the study after valproic acid dosing was decreased and CHE was continued at 10–12 mg/kg/day.

No clinically significant adverse events directly attributed to the CHE were encountered. Two participants had serious adverse events requiring hospitalization, but these were not related to the study drug. During their hospitalizations, both remained on their routine anticonvulsants and CHE.

### Efficacy Outcome Measures

The average reduction in daily seizure frequency between visits for each participant is displayed in Figure 1A. Over the study period, all seven participants had an improvement in seizure frequency with CHE. One participant (A-04) had a transient worsening of seizures at a CBD equivalent dose of 2–3 mg/kg/day. All participants had a reduction in average daily seizure frequency at a CBD equivalent dose of 5–6 mg/kg/day with six participants having a decrease >25% and four participants having a decrease >50%. After increasing to 10–12 mg/kg/day, the average reduction across all participants was 74% (Figure 1B) with all participants having a >25% reduction in daily seizure frequency, five participants having a decrease >50%, and three participants being seizure free. One participant was seizure free on an 8–9 mg/kg/day CBD equivalent dose.

During the final month of the study, when CHE was weaned off completely in the first three weeks, the reduction in seizure frequency was maintained in all participants and continued to improve in three participants (A-02, A-04, A-05) despite no changes to their anticonvulsant regimens.

While there was a reduction in daily seizure frequency between visits for all seizure types recorded, the greatest reduction was seen in atonic and versive seizures while epileptic spasms increased in frequency (Figure 1C). The percentage reduction in frequency of reported seizure types compared to baseline for all seven participants at each visit are also provided as Supplementary Figures 1A–G.

By the time the CBD dose was increased to 10–12 mg/kg/day, all participants -except for participant A-07, who had a normal background activity on the initial EEG– had an improvement in their EEG encephalopathy rating scale with most improving by one point on the rating scale. Participant A-03 had an improvement by two points. During the course of the study, three participants had an improvement in their EEG Spike Index scores. Participants A-03 and A-04 had resolution of their continuous spike activity in sleep. Full details of EEG results are provided in Supplementary Figures 2A,B.

An improvement in the total QOLCE-55 scores was observed in all participants with the greatest improvements were found on the Cognitive, Social and Emotional Functioning subscales (Figure 2). While the improvements in the QOLCE scores decreased during the weaning period following Visit 6 the scores remained improved over the baseline scores.

### Plasma Cannabinoid Plasma Levels in Relation to Dosage Escalation and Decrease in Seizure Frequency

Cannabinoid C_SS, Min_ plasma concentrations were measured at the end of each subsequent month's dosage escalation (Figures 3A–F). With each dosage escalation, CBD and CBC C_SS, Min_ values generally increased proportionally with dose in all participants, except for participant A-04, whose last dose escalation resulted in non-proportional increases in both CBD and CBC C_SS, Min_ values (Figures 3A–C).

Following a month of CBD at 5–6 mg/kg/day, the four participants with a >50% reduction in average daily seizure frequency, had C_SS, Min_ CBD levels ranging from 14.8 to 24.4 ng/mL. After a month of CBD at 10–12 mg/kg/day, the five participants with a >50% reduction in average daily seizure frequency, had C_SS, Min_ CBD level ranging from 42.5 to 124.7 ng/mL. The C_SS, Min_ CBD levels corresponding with the CBD dosage at which the three participants became seizure free, ranged from 54.8 to 78.9 ng/mL (Figure 3A).

In all but two participants (A-04 and A-07), C_SS, Min_ THC levels were detectable at 2–3 mg/kg/day. Even at the highest dose of CHE, the C_SS, Min_ THC levels were low—below 4 ng/mL in all but two participants with the highest level being 4.34 ng/mL (Figure 3E).

### Effect of CHE on Steady State Levels of Anticonvulsants

Apart from clobazam, C_SS, Min_ anticonvulsant levels did not change significantly and remained within therapeutic limits with the following exceptions. Valproic acid levels for participant A-07 doubled between visits 2 and 6 but remained within therapeutic range (350–700 umol/L). For participant A-06, Valproic acid levels decreased to below therapeutic range at 16 umol/L between visits 4 and 5 suggesting medication non-compliance. C_SS, Min_ clobazam and norclobazam levels of the four participants taking clobazam during the study are provided in Supplementary Table 2. For participants A-02 and A-03 these levels are not available for visit 6 due to the samples being misplaced in our hospital central laboratory. Three participants (A-01, A-02, and A-03) experienced side effects felt to be secondary to clobazam prompting a decrease in their clobazam dosage. In all three participants, these apparent side-effects of clobazam resolved with a decrease in clobazam dosing. Co-administration of clobazam did not appear to correlate with higher levels of CBD or CBC at each dosage escalation.

## Discussion

CARE-E is an open label dosage finding study designed to assess the safety and efficacy of a CBD-Enriched *Cannabis* Herbal Extract (CHE) in children with intractable epileptic encephalopathy. The study involved measurement of C_SS, Min_ levels of CBD, THC, and CBC and their relationship with safety, tolerability, and efficacy outcome measures in hopes to identify appropriate doses of similar *Cannabis* products in children. This study is the first to report pediatric C_SS, Min_ levels of CBD, THC, and CBC in any pediatric dosage escalation study and provide guidance on initial dosing of CBD-enriched CHEs.

Escalating doses of CBD-enriched CHE from 2–3 mg/kg/day to 10–12 mg/kg/day resulted in no serious adverse events related to the CHE. Parents reported sleepiness/lethargy and irritability in most participants, but these side effects were assessed after starting the study drug and were likely pre-existing. Transient increases in sleepiness and irritability in three participants taking clobazam resolved after clobazam dose was decreased, suggesting these side-effects could be secondary to an interaction between the CHE and clobazam (14). Laboratory monitoring noted significant elevations in GGT, AST, and lipase levels in two participants, both of whom were also taking valproic acid. Participant A-03's marked elevation of GGT was likely secondary to sepsis, which occurred during the study. Participant A-04's transient and non-significant elevation of AST and significant elevation of lipase levels were likely secondary to a predisposition to hepatic and pancreatic dysfunction from high-dose valproic acid and corroborates observations reported elsewhere (3, 16). The fact that this participant had a preexisting elevated GGT suggests that liver enzymes should be screened prior to starting CHE, especially if the child is already prescribed valproic acid.

The concentrations of CBD, THC, and CBC appeared to increase linearly with dosage in six of the seven participants, suggesting dose-independent pharmacokinetics for these participants within this dosage escalation trial. The greater than proportional increase in C_SS, Min_ CBD with the final dosage increase in participant A-04 may suggest dose-dependent pharmacokinetics with saturation of first-pass metabolism and an increase in the oral bioavailability. Participant A-04 did not exhibit any change in clinical status or in anticonvulsant therapy to explain this disproportional increase in C_SS, Min_. To confirm the possible non-linear pharmacokinetics in children, a dosage escalation study involving a larger sample size and a higher dose beyond the doses used in the current trial will be necessary. The possibility of dose-dependent PK, though, raises a safety concern, which also warrants further investigation in pediatric patients and suggests a need to limit dose sizes and not to simply continue increasing doses until an appropriate effect is observed.

CBD and THC both inhibit enzymes involved in the metabolism of many anticonvulsants including CYP2C and CYP3A isoenzymes (17). While increases in Clobazam and norClobazam levels were seen in some participants taking clobazam, overall co-administration of CHE did not significantly affect C_ss, min_ levels of the other concomitant anticonvulsants. It would have been of interest to measure C_ss, min_ levels of stiripentol given that many children with Dravet syndrome would also be taking this medication and stiripentol is metabolized by CYP2C19 and CYP3A4 isoenzymes. At present it is unknown if there is a pharmacokinetic interaction between CBD and Stiripentol. Although an assay to measure stiripentol levels is described, it is not indicated for therapeutic purposes by the manufacturer or regulatory bodies such as Health Canada, FDA or the EU. As such, it was not available for us for the purposes of this study (18).

The potential intoxicating effects of any THC present in CHE remain a concern for pediatric patients. Oral consumption of *Cannabis* products results in lower peak levels of THC as compared to smoking due to a high first-pass effect and slow erratic absorption from the gastrointestinal tract. However, intoxication can still occur because of greater distribution into the central nervous system and conversion to 11-hydroxy-THC, which is also intoxicating and has a half-life as long as, or longer, than THC (17, 19, 20). The C_SS, Min_ levels of THC increased in a seemingly linear relationship to dosage, and with the exception of two participants at the highest dosage level, these remained lower than levels that have been reported to cause intoxication (19). Tachycardia and conjunctival injection—felt to be reliable markers of intoxication from THC—were not seen during the study. The lack of intoxication seen in our participants whose plasma THC levels exceeded 4 ng/mL may have been due to the reported CBD-mediated attenuation of the intoxicant effects of THC (21).

An overall trend for improvement in seizure control and QOLCE scores was observed with increasing CHE dosage and C_SS, Min_ CBD levels. A >50% reduction in average daily seizure frequency occurred in four of seven participants at a CBD dose of 5–6 mg/kg/day, and all participants had a >25% reduction in seizures at a CBD dose of 10–12 mg/kg/day. In the QOLCE scores, there was a trend toward improvements in cognitive, social, and emotional function in relation to CBD dosage. These data suggest that the initial target dose of CBD should be 5–6 mg/kg/day when a 1:20 THC:CBD whole plant extract is used and can be increased as needed up to 10–12 mg/kg/day with careful consideration of potential non-linear pharmacokinetics at higher doses.

Trembly and Sherman reported that adult patients taking purified CBD had no improvement in seizure control when their plasma CBD levels ranged from 20 to 30 ng/mL, while a significant decrease in seizure control occurred when plasma CBD levels increased above 150 ng/mL (22). While it is challenging to correlate C_SS, Min_ levels of CBD and CBC with efficacy in reducing seizure frequency based on our data, we do note that the C_SS, Min_ CBD levels associated with a >50% reduction in average daily seizure frequency and seizure freedom in this study were lower. Further analysis with larger sample sizes are needed to delineate which C_SS, Min_ level of CBD is associated with optimal seizure control and improved QOLCE scores.

Two recent systematic reviews of clinical trials assessing pharmaceutical grade CBD in children with treatment epilepsy provide insight into the expected outcomes at the CBD doses used in these trials. In pooled data of 17 observational studies, Stockings et al. found that CBD at 20 mg/kg/day resulted in 48.5% of patients having a 50% reduction in seizures and QoLCE scores improved in 55.8% (23). Lattanzi et al. also performed a systematic review of the four clinical trials assessing pharmaceutical grade CBD in children with treatment resistant Lennox Gastaut and Dravet Syndromes. They reported that the pooled average difference in seizure frequency between CBD and placebo with CBD at 10 mg/kg/day was 19.5% while that with CBD at 20 mg/kg/day was 19.9% both in favor of CBD. A seizure frequency reduction of 50% (for all seizure types) was 37.2% with CBD at 20 mg/kg/day and 21.2% with placebo (24).

An “entourage” effect in which the clinical efficacy of cannabinoids when used in combination are greater than when used individually has been demonstrated in several animal models of epilepsy but has yet to be reported for human trials (25–27). While we saw clinical efficacy with regards to reduction in seizure frequency and improvements in QoL scores with CBD doses lower than those reported in studies using pharmaceutical grade CBD, the small number of participants reported require caution when interpreting the results and preclude drawing definite conclusions in particular with regards to possible entourage effect. Additionally, CARE-E was not designed to compare efficacy of CHE to pharmaceutical grade CBD. This can only be addressed in a head to head comparative study.

The preliminary findings presented in this manuscript are however, in keeping with the results of a metanalysis of clinical studies comparing whole plant Cannabis CHE to pharmaceutical grade CBD in children with refractory epilepsy. This metanalysis found that while there was no significant difference between the CHE and pharmaceutical grade CBD in attaining 50% reduction in seizures, 71% of children taking CHE had improvement in seizure frequency compared to 46% taking purified CBD (*p* < 0.0001). The average CBD dose for children taking CHE was 6 mg/kg/day (28).

The three participants who became seizure free were taking long acting benzodiazepines (clobazam or clonazepam) but clobazam and clonazepam levels did not increase for two of these participants. This suggests that, while CBD and long acting benzodiazepines likely have a synergistic effect, this is not necessarily due to an increase in plasma benzodiazepine levels.

The reported half-life of CBD ranges from 9 to 32 h; however, the influence of age and concomitant anticonvulsants on the half-life remain largely unknown (29). These influences on half-life, however, would not explain the month-long continued improvements in seizure control and QOLCE scores observed in our three participants during the wean-off CHE. Such sustained effects often involve epigenetic changes and therefore it is possible that any long-term beneficial effect of CBD may reflect, in part, an as-of-yet unrecognized epigenetic effect (30). In order to assess if any long-lasting effect might be mediated through an active metabolite of CBD, we will measure participants' plasma levels of CBD, CBC, THC, and their metabolites upon completion of the 1-month weaning period in subsequent participants who are enrolled in CARE-E.

While an improvement in background EEG activity was seen in four participants, there was no correlation between CBD dosing and improvements on the background score. A more complete examination of a potential relationship between the improvement in cognitive functioning seen in the QOLCE scores and improved background activity seen on EEG is warranted. The reduction in spike count following CBD treatment in three participants is reminiscent of the effect observed with broad-spectrum anticonvulsants such as benzodiazepines and valproic acid (31).

Although the reported improvement in seizure control and quality of life are promising, these findings must be interpreted with caution as there are several limitations in this preliminary report, in particular the small sample size and potential reporting bias inherent with open label studies. The lack of a placebo group and self-reporting of outcomes may limit the ability to discern any placebo effect which is seen in many drug trials. Reporting fatigue experienced by caregivers also may be a confounder, in particular for seizure frequency data.

## Conclusion

The preliminary results of seven participants from the CARE-E study suggest CBD-enriched CHE up to 10–12 mg/kg/day is generally well tolerated. All participants had improvements in seizure frequency, modified Quality of Life in Childhood Epilepsy (QOLCE), and electroencephalogram (EEG) rating scores. Steady state C_SS, Min_ data for CBD, THC, and CBC suggest linear PK, although one participant gave possible evidence of non-linear PK at higher doses. The preliminary data suggest an initial CBD target dose of 5–6 mg/kg/day when using a 1:20 THC:CBD CHE in children with treatment resistant epileptic encephalopathy. C_SS, Min_ CBD levels suggest that dosing with a CHE containing THC and other cannabinoids may be more effective than purified CBD alone. Based on clinical observations and measurement of plasma THC levels, intoxication from THC is unlikely to occur when a 1:20 THC:CBD CHE is used within therapeutic doses. The anticonvulsant effect of CHE persisted after it was weaned off, suggesting an enduring anticonvulsant effect.

## Data Availability

The datasets generated for this study are available on request to the corresponding author.

## Ethics Statement

This study was carried out in accordance with the University of Saskatchewan Biomedical Research Ethics Review Board with written informed consent from the parents/legal guardians of all subjects. The parents/legal guardians of all subjects gave written informed consent in accordance with the Declaration of Helsinki. The protocol was approved by the University of Saskatchewan Biomedical Research Ethics Review Board.

## Author Contributions

RH, RT-W, JA, BA, SC, RL, AL, SM, DM, DN, EP-L, BS, and JT-Z contributed to the design of the study protocol. RH, LH, EL, and PM are site investigators for CARE-E. SC analyzed the data. RH, RT-W, JA, RL, and AL interpreted the data. SV assisted with the development and validation of the plasma cannabinoid assay used in this study. RH drafted the manuscript. All authors contributed to the revision of the manuscript and approved it for submission.

### Conflict of Interest Statement

The authors declare that the research was conducted in the absence of any commercial or financial relationships that could be construed as a potential conflict of interest.
