# Supplementary material for: Dosage Related Efficacy and Tolerability of Cannabidiol in Children With Treatment-Resistant Epileptic Encephalopathy: Preliminary Results of the CARE-E Study
Source: Front Neurol. 2019 Jul 3;10:716. doi: 10.3389/fneur.2019.00716 (PMC6616248; doi:10.3389/fneur.2019.00716)
Supplement: Supplementary file 1 [file Table_1.pdf]

Supplementary Tables 1a to 1d

Supplementary Table 1a: Participant Sleepiness/Lethargy Side Rating Scores

Participant	Visit				
	3	4	5	6	7
**A-01**	1	0	0	0	0
**A-02**	0	0	0	0	0
**A-03**	2	1	1	3	1
**A-04**	1	1	2	1	1
**A-05**	1	0	1	2	1
**A-06**	1	2	1	2	1
**A-07**	0	1	0	1	1

Supplementary Table 1b:Participant Irritability Rating Scores

Participant	Visit				
	3	4	5	6	7
**A-01**	3	3	2	3	2
**A-02**	0	0	1	0	2
**A-03**	2	2	1	3	1
**A-04**	2	1	3	3	1
**A-05**	3	2	1	3	2
**A-06**	1	1	1	1	1
**A-07**	1	0	1	1	2

Supplementary Table 1c: Participant Nausea/Vomiting Rating Scores

Participant	Visit				
	3	4	5	6	7
**A-01**	0	0	0	0	0
**A-02**	0	0	0	0	0
**A-03**	1	0	0	4	0
**A-04**	1	2	0	3	1
**A-05**	0	0	0	1	0

**A-06**	0	0	0	0	0
**A-07**	0	0	0	0	2

Supplementary Table 1d: Participant Diarrhea Rating Scales

**Participant**	**Visit**				
	**3**	**4**	**5**	**6**	**7**
**A-01**	0	0	0	1	0
**A-02**	0	0	0	0	0
**A-03**	0	0	0	0	0
**A-04**	0	0	0	2	0
**A-05**	0	0	1	3	2
**A-06**	1	1	0	0	0
**A-07**	5	0	2	1	4
