# Supplementary material for: Dosage Related Efficacy and Tolerability of Cannabidiol in Children With Treatment-Resistant Epileptic Encephalopathy: Preliminary Results of the CARE-E Study
Source: Front Neurol. 2019 Jul 3;10:716. doi: 10.3389/fneur.2019.00716 (PMC6616248; doi:10.3389/fneur.2019.00716)
Supplement: Supplementary file 2 [file Table_2.pdf]

Supplementary Table 2: Supplemental Table 2 | Clobazam dosage (in mg/kg/day), Clobazam (CBZ), and norclobazam (norCBZ) Css,Min plasma concentrations for the four participants taking clobazam in conjunction with CHE. Levels at Visit 6 for participants A-02and A-03 are not available (N/A) for analysis. Also included are reported side effects attributed to an interaction between clobazam and CHE and the resultant changes clobazam doses. All participants had resolution of their reported side effects with decreased clobazam dosing.

Participant	Visit	CBZ dosage (mg/kg/day)	CBZ level (nmol/l) Reference (150-1000 nmol/l)	norCBZ level (nmol/l) Reference (2800-14,000 nmol/l)	Side effect reported	Action Taken
A-01	2	1.2	4038	44798	-	-
	3	1.2	4327	62022	Excessive sleepiness	Decrease clobazam to 1 mg/kg/day
	4	1.0	2894	45096	-	-
	5	1.0	1835	24298	-	-
	6	1.0	1545	24230	-	-
A-02	2	0.3	687	3486	-	-
	3	0.3	286	3108	-	-
	4	0.3	133	1949	Increased Irritability	Decrease clobazam to 0.15 mg/kg/day
	5	0.15	91	779	-	-
	6	0.15	N/A	N/A	-	-
A-03	2	1.2	747	2143	Increased sleepiness after visit 2	Decrease clobazam to 0.8 mg/kg/day
	3	0.8	1145	5892	-	-
	4	0.8	1068	11881	-	-
	5	0.8	812	6317	-	-
	6	0.8	N/A	N/A	-	-
A-07	2	1.1	1840	5350	-	-
	3	1.1	1550	12080	-	-
	4	1.1	<50	5850	-	-
	5	1.1	920	16320	-	-
	6	1.1	760	24440	-	-
