# Supplementary material for: Dosage Related Efficacy and Tolerability of Cannabidiol in Children With Treatment-Resistant Epileptic Encephalopathy: Preliminary Results of the CARE-E Study
Source: Front Neurol. 2019 Jul 3;10:716. doi: 10.3389/fneur.2019.00716 (PMC6616248; doi:10.3389/fneur.2019.00716)
Supplement: Supplementary file 3 [file Data_Sheet_1.docx]

**Supplemental Protocol: Details of Quantification of Plasma Cannabinoids**

*Chemicals, Reagents, and Supplies*

CanniMed^®^ Oil 1:20 (1 mg/mL of Δ^9^-THC and 20 mg/mL of cannabidiol (CBD), an oil-based extract of *Cannabis sativa,* was purchased from CanniMed^®^ Therapeutics Incorporated. Analytical standards of CBD, Δ^9^-THC, and cannabichromene (CBC) were purchased from Cerilliant at a concentration of 1 mg/mL, dissolved in methanol. CBD-d3, Δ^9^-THC-d3, and CBC-d9 internal standards were also purchased from Cerilliant at a concentration of 0.1 mg/mL, dissolved in methanol. All stock and internal standards were stored at -20°C until use. Optima^TM^ LC-MS grade water, acetonitrile, and methanol were purchased from Fisher Scientific. For blood collection, BD Vacutainer^®^ Barricor™ tubes were provided by BD Biosciences. Eppendorf™ Protein LoBind microcentrifuge tubes were obtained from Fisher Scientific. Human blank plasma (lithium heparin) was obtained from BioreclamationIVT. Double deionized water was provided from a MilliQ Synthesis Water Purification system. All other solvents were LC/MS grade and all other chemicals were reagent grade.

*Quantification of Cannabinoids in Plasma*

The plasma samples (200 µL) were prepared and analyzed according to a validated liquid chromatography-mass spectrometry method described in the CARE-E protocol paper [14]. Briefly, stock solutions in methanol (1 mg/mL) of cannabinoids and their respective stable isotope labeled internal standards were serially diluted with blank human plasma to produce working solutions for the calibration curves. The calibrations curve range for CBD and Δ^9^-THC, were 0.49 ng/mL and for CBC 0.98 ng/mL. Sample preparation consisted of the addition of 610 μL of cold acetonitrile spiked with internal standard (1.6 ng/mL of each deuterated internal standard) to 200 μL of plasma sample in microcentrifuge tubes. Samples were vortex mixed for 10 seconds, centrifuged for 10 min at 14000 rpm in a microcentrifuge set at 4°C, and 700 μL of supernatant transferred to clean glass tubes and dried with filtered air at 35°C and protected from light for 20 minutes. Samples were reconstituted with 200 μL of mobile phase, vortex-mixed for 20 seconds, and transferred to HPLC inserts in amber autosampler vials for mass spectrometric analysis. Quality control (QC) standards were prepared similarly and acceptance criteria for each analytical run was based on low, medium, and high concentration QC standards. The lowest QC for CBD, THC, and CBC were 1.4 ng/mL. The middle QC and highest QC for all cannabinoids were 50.56 ng/mL and 101.2 ng/mL, respectively.

For LC-MS/MS analysis, 5 μL was injected onto a Zorbax Eclipse XDB-C18 Narrow-Bore 2.1 x 75 mm 3.5-micron column with a Zorbax Eclipse XDB-C8 Narrow-Bore 2.1 x 12.5 mm 5-micron guard column, both controlled at 30°C, using gradient mobile phase conditions. Mobile phase A consisted of LC/MS grade water containing 0.1 mM ammonium formate and mobile phase B consisted of LC/MS grade methanol containing 0.1 mM ammonium formate and gradient conditions were 0-3.5 min 20A:80B, 3.5-10 min 10A:90B, 10-10.5 min 20A:80B, and 10.5-13.5 min 20A: 80B. The flow rate was 250 μL/min with a run time of 13.5 minutes, and the autosampler was set at 4°C. Samples were separated using an Agilent 1290 Infinity LC System and analytes were detected with an SCIEX QTrap^®^ 6500 system. The mass spectrometer was set to positive ion polarity mode. The QTRAP 6500 utilized a curtain gas pressure of 10 pse, and GS1 and GS2 parameters were set at 50 psi. The ionspray voltage was set at 4500 V and the temperature of the ESI source interface was maintained at 650°C. The multiple reaction monitoring conditions are outlined in Table 1. SCIEX Analyst^®^ 1.6.2 was used to perform instrumental control and data analysis. SCIEX MultiQuant^™^ 3.0.1 was used to perform quantitation analysis. The ratio of the peak areas of the cannabinoids to their respective internal standard were plotted against the nominal concentrations to construct the calibration curve and a linear least-squares regression analysis using 1/X as weighting factor determined the slope, intercept, and coefficient of determination (*r*^2^) to demonstrate linearity of the method. Sample concentrations were interpolated from the calibration curve. Analytical method validation for the cannabinoids was performed in accordance with USDFA guidelines.
