# Supplementary figures and images for: Dosage Related Efficacy and Tolerability of Cannabidiol in Children With Treatment-Resistant Epileptic Encephalopathy: Preliminary Results of the CARE-E Study
Source: Front Neurol. 2019 Jul 3;10:716. doi: 10.3389/fneur.2019.00716 (PMC6616248; doi:10.3389/fneur.2019.00716)

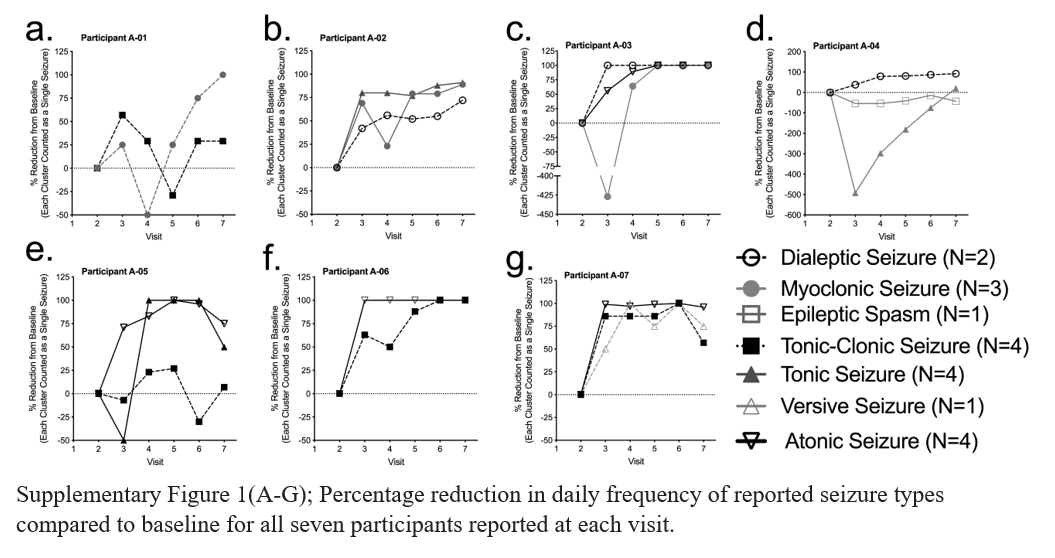

Supplement: Supplementary file 4 [file Image_1.tiff]

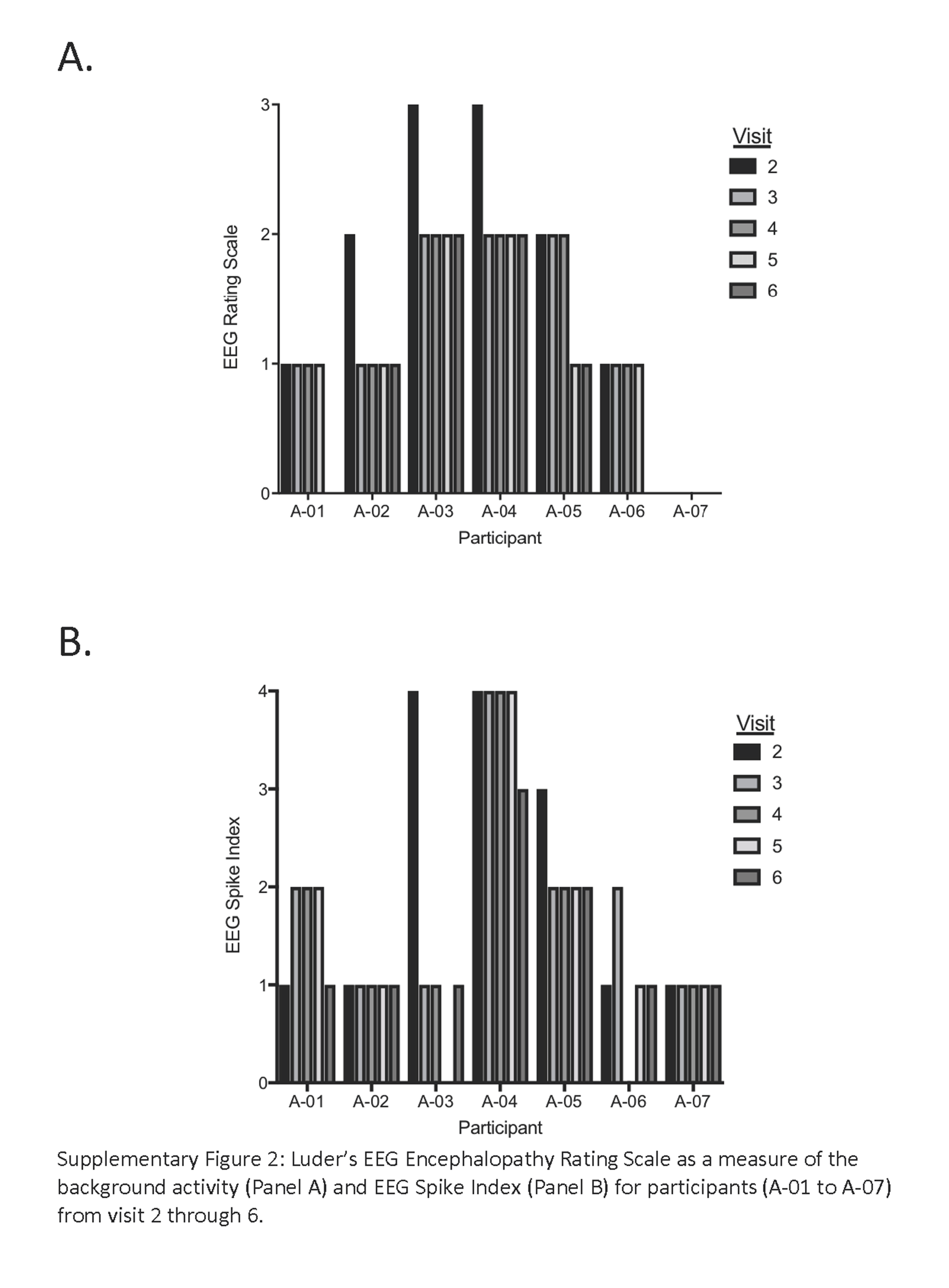

Supplement: Supplementary file 5 [file Image_2.tiff]
